# Exploring the Impact of the Caring Contacts Intervention on the Stress and Distress of Veterans and Service Members: Protocol for a Randomized Controlled Trial

**DOI:** 10.2196/72140

**Published:** 2025-08-13

**Authors:** Barbara Wright, Anna Evanson, Cameron Casey, Keyne C Law, Andrew H Rogers, Katherine Anne Comtois

**Affiliations:** 1 Department of Psychiatry & Behavioral Sciences School of Medicine University of Washington Seattle United States; 2 Department of Clinical Psychology School of Psychology, Family, and Community Seattle Pacific University Seattle United States; 3 Division of Behavioral Medicine Department of Medicine University at Buffalo, State University of New York Buffalo United States; 4 Department of Pediatric Oncology Roswell Park Comprehensive Cancer Center Buffalo, NY United States

**Keywords:** suicide, intervention, stress, RCT, randomized controlled trial, EMA, ecological momentary assessment, Caring Contacts

## Abstract

**Background:**

Suicide is a recognized global public health problem that continues to pose substantial challenges in the United States. Veterans and service members are particularly at risk. Caring Contacts is a simple, scalable intervention comprising brief, periodic messages sent over 1 to 2 years that express unconditional care and concern. It reduces suicide risk among individuals with recent suicidal ideation or attempts and has demonstrated acceptability in military and veteran samples, but little is known about the mechanisms of Caring Contacts or its applicability to populations who are not suicidal.

**Objective:**

We aim to evaluate if receiving Caring Contacts reduces suicide risk among veterans and active service members recruited based on their stress or distress levels, using ecological momentary assessment (EMA) and survey measures of suicidal ideation and cognitions; to examine the experiences of diverse veterans and service members with Caring Contacts; to evaluate if receiving Caring Contacts reduces distress (depression, substance use consequences, hopelessness, defeat, and psychological pain); and to identify the potential mechanisms of action for Caring Contacts (mattering, connectedness, social responsibility, and entrapment). Using EMA, this study will be the first to investigate mechanisms of the Caring Contacts intervention both in real time, as the messages are received, and over the course of 1 year.

**Methods:**

In this randomized controlled trial, veterans or service members (N=510) experiencing stress or distress recruited via social media advertisements will be offered best available resources and randomized into 1 of 3 study conditions: 12 months of Caring Contacts and monthly EMA, 12 months of Caring Contacts without monthly EMA, or 12 months of monthly EMA alone. The individuals in the Caring Contacts conditions will receive 13 messages. A subset of participants from each condition will be asked to complete a qualitative interview after the 12-month follow-up about their study experience.

**Results:**

This trial was funded in October 2023; recruitment started in April 2024 and will conclude in May 2025. As of December 2024, we have enrolled 321 participants. Final quantitative data collection will be completed by July 2026, 2 months after the final 12-month follow-up date. Data from the qualitative interviews will be collected until September 2026. Data analysis will occur following data collection, and the results are expected to be published in winter 2027.

**Conclusions:**

This study will be the first randomized controlled trial evaluating the impact of Caring Contacts among service members and veterans who were not recruited based on suicidality. If effective, our findings will demonstrate that Caring Contacts is beneficial for reducing multiple forms of psychological distress and suicide risk. In addition, we will evaluate potential mechanisms explaining the effects of Caring Contacts and assess the utility of EMA as a primary measure of outcome in suicide research trials.

**Trial Registration:**

ClinicalTrials.gov NCT06136234; https://clinicaltrials.gov/study/NCT06136234

**International Registered Report Identifier (IRRID):**

DERR1-10.2196/72140

## Introduction

### Background

According to the Centers for Disease Control and Prevention, 1 person in the United States died by suicide every 11 minutes in 2022, and suicide rates have substantially increased over the past decade [1]. However, mortality rates from heart disease, diabetes, and cancer have declined. Since 2011, new standards have led to improved screening and management of suicide risk [2-7].

However, treatment to reduce suicide risk has lagged because of the high cost of most evidence-based suicide care interventions, which require multiple sessions of psychotherapy [3,8-10]. Thus, the need for suicide prevention has skyrocketed without a corresponding increase in available resources. In addition, the COVID-19 pandemic substantially increased the demand for telehealth and technology-mediated interventions—a trend that has persisted, with both patients and health care providers continuing to favor remote treatment options [11-13]. Thus, there is a pressing need to quickly adopt scalable alternatives to office-based care for suicide prevention.

The Caring Contacts intervention consists of brief, periodic messages sent over 1 to 2 years that express unconditional care and concern. It has shown promise in reducing suicide, suicide attempts, and suicide ideation in at least one randomized trial [14-20]; is cost-effective [21]; is recommended by multiple clinical practice guidelines [4,5]; and has the potential to scale with a single individual messaging with hundreds of suicidal individuals simultaneously. A meta-analysis of Caring Contacts concluded that it has a protective effect against suicide attempts [22]. However, findings on the intervention’s impact on suicidal ideation (SI), suicide deaths, and health care use— such as emergency department visits or hospitalizations—have been mixed [22,23]. While some fully powered recent studies have shown no substantial effects [24,25], others suggest that Caring Contacts decreases SI [18,20] and reduces the number of medical and psychiatric hospitalizations during follow-up [16,17]. These mixed findings underscore the importance of understanding the mechanisms through which Caring Contacts achieves its effects, as this knowledge could guide improvements to Caring Contacts as well as improve implementation by understanding why it works in some contexts but not in others. In previous research, our team has adapted Caring Contacts for SMS text messages and has culturally adapted and implemented Caring Contacts for military populations [20], Native communities [25], and through fully online trials in Idaho’s rural and frontier health systems [26]. Colleagues have developed Caring Contacts models for veterans receiving Veterans Affairs services [27-31]. In addition, we have demonstrated that the intervention is both feasible and acceptable when delivered by community laypersons without clinical training [25]. Therefore, Caring Contacts is well-positioned for scale-up with veteran service organizations, with one significant limitation that it has only been evaluated with individuals who are seeking help due to suicidality, self-harm, or severe depression. It is unknown if Caring Contacts is helpful or suicide preventive for a broader population of veterans experiencing stress or distress. This is crucial because a large proportion of veterans who die by suicide do not disclose suicide risk and are served by veteran service organizations that do not screen for suicidality, so their risk is unknown [32]. As Caring Contacts do not discuss suicidality explicitly, there is no reason that the intervention should only apply to those experiencing suicidality. Further research is needed to determine whether Caring Contacts can reduce suicide risk in a broader population and how the intervention is experienced by individuals who are acutely suicidal compared to those who are not. In addition, it is possible and desirable to explore whether Caring Contacts can impact other forms of psychological distress. We aim to address these key gaps in Caring Contacts research.

While Caring Contacts has shown promise as a suicide prevention strategy, understanding its mechanisms of action is essential for refining and optimizing its effectiveness. Currently, these mechanisms remain unknown, as most research on Caring Contacts has focused on administrative records for assessment or contact only before and after the intervention [15,16,19,20,24,33,34]. It is often theorized that the Caring Contacts intervention reduces suicide risk by fostering a sense of connection, as individuals may feel cared for and less isolated after receiving messages of support [15,35]. However, this theory has not been formally tested. To evaluate whether the Caring Contacts intervention decreases SI by enhancing connectedness, we selected 4 mechanisms to examine in this study: perceived burdensomeness, mattering, personal and social responsibility, and entrapment. Perceived burdensomeness has consistently been identified as a robust predictor of SI, reflecting the damaging impact of feeling like a burden to others. Mattering reflects the perception of being valued and important, directly countering feelings of insignificance and isolation, which have been linked to suicide [36,37]. Although a sense of personal and social responsibility has not been explicitly examined as a predictor of suicide, it may encourage individuals to engage with broader social systems [38], thereby strengthening their sense of belonging [39] in their communities. Finally, entrapment captures the psychological state of feeling trapped with no escape, which is strongly associated with SI and intent within the integrated motivational-volitional model of suicidal behavior [40-42]. Together, these mechanisms encompass both interpersonal and intrapersonal dimensions of connectedness, offering a comprehensive framework for understanding how Caring Contacts may decrease suicide risk.

To understand the mechanisms and core processes driving the effectiveness of Caring Contacts, it is crucial to be able to capture the dynamic and evolving nature of outcomes over time. Traditional pre- and postintervention assessments often fail to account for the nuanced, day-to-day, or even rapid fluctuations in SI, stress, and distress [43]. This study will be the first to use periodic ecological momentary assessment (EMA) during a study of the Caring Contacts intervention to capture how the intervention decreases SI, stress, and distress. EMA is an intensive longitudinal data collection method that involves repeatedly sampling participants’ current behaviors and experiences in real time in participants’ natural environments, thereby reducing the chance of recall bias while also separating how much of the process is attributable to person- or sample-specific microprocesses that influence behavior in real time [29,30]. In addition, given the frequency of assessments, we are able to examine variability in constructs of interest over a discrete EMA period. EMAs are particularly important in suicide research given the dynamic, fluctuating nature of SI and its associated risk factors. In suicide research, EMA is particularly valuable for tracking the emergence of SI and tracking its dynamic nature along with its proximal warning signs, drivers, and risk factors [44,45]. Indeed, greater SI has more often been reported on EMAs than retrospective reports such as surveys.

Furthermore, EMA-based models have demonstrated strong predictive accuracy for near-term suicidal thoughts and posthospital suicide attempts [31,32], highlighting the utility of self-reported data and real-time monitoring in guiding risk assessment and intervention delivery. As such, using EMA to study time-varying changes in suicide risk and related proximal risk factors has been a priority for the advancement of suicide research [43,46]. However, EMA has been underused to study suicide risk in veterans and service members despite its feasibility [47] and potential for increasing understanding of mental health problems [48]. By using EMA as our primary outcome measure, we will explore how Caring Contacts influences proximal risk factors and mechanisms of change in veterans and active-duty service members, both when individual messages are received and across the year-long intervention period. To control for the potentially confounding impact of frequent EMA and the study intervention, 3 conditions are used to study Caring Contacts both with and without monthly EMA.

### Aims

Our primary aim in this study is to evaluate whether stressed or distressed veterans and active-duty service members experience a greater reduction in suicide risk, suicide urges, the wish to live, and the wish to die when receiving Caring Contacts. We will measure these outcomes through EMA and survey measures of SI and cognitions. The second aim is to examine the experiences of diverse veterans and service members with Caring Contacts (ie, access, satisfaction, and preferences) via qualitative interviews. The third aim is to evaluate if veterans and service members receiving Caring Contacts will show reductions in measures of distress (ie, isolation, depression, substance misuse, loneliness, defeat, hopelessness, and psychological pain). Finally, the fourth aim is to examine potential mechanisms of action for Caring Contacts (ie, mattering, connectedness, social responsibility, and entrapment) measured via EMA.

## Methods

### Study Design

In this randomized controlled trial (RCT), we will examine the impact of the Caring Contacts intervention delivered through SMS text message across a large, diverse sample of veterans and service members who are stressed (external factors) or distressed (internal factors). This trial is conducted entirely online and will recruit veterans and service members (N=510). To ensure that all participants experiencing stress or distress have access to needed resources (enhanced usual care), each participant, regardless of condition, is provided a tailored set of resources to match their needs (eg, housing, finances, employment, food insecurity, and socialization). Participants are stratified based on whether they endorsed SI in the past year and randomly assigned to 1 of 3 study conditions, including receiving either Caring Contacts, EMA, or both, for the 12-month study period. Given that the frequency of messages and the self-monitoring process inherent to EMA may have its own effect on our outcome variables, we included a Caring Contacts–only condition to isolate the effects of Caring Contacts and EMA. The study design and methods were evaluated during an acceptability and feasibility pilot study, during which we also developed a web-based app platform custom-made for Caring Contacts (manuscript in preparation). The study staff worked with employees housed within a local veteran service organization and an outpatient psychiatry clinic to develop study procedures and the Caring Contacts intervention. From this feedback and use data, adjustments to the protocol, intervention, and EMA procedures were made before the launch of this RCT.

### Setting

The study protocol is designed to operate entirely in an internet-based environment, using web-based recruitment and remote study staff. The study staff selection prioritized researchers with extensive experience overseeing large virtual clinical trials and implementing the Caring Contacts intervention. In addition, the research team is housed within a suicide specialty research center at the University of Washington (UW), where staff receive ongoing continuing education about suicide research.

### Recruitment

Recruitment began in April 2024 and is anticipated to continue until May 2025. The data collection is ongoing and will continue until July 2026. Participants are primarily recruited using Nativve Health Research, a UK-based company that specializes in digital advertising for clinical research. Recruitment is primarily conducted through advertisements on Meta’s platforms (including Facebook and Instagram), although advertisements on Google and X (previously known as Twitter) were used early in the study but were discontinued as they were less fruitful. Marketing of advertisements is segmented as needed to achieve demographic diversity of age, race or ethnicity, gender identity, education, regional representativeness, and rurality.

To mitigate the risk of fraud, the social media advertisement does not mention financial compensation. In addition, each participant is verified using a combination of fraud prevention strategies. To minimize the risk of fraudulent participation by automated bots, honeypots—hidden fields visible only to bots—were programmed into the study flow. Other fraud reduction techniques include outliers for the time it took for a participant to complete the survey, not sharing the participation link publicly, and attention check questions [49].

### Ethical Considerations

#### Human Participant Ethics Review Approvals

The UW is the sponsor, and the UW Institutional Review Board (IRB) approved the study protocol and all modifications under the study identification number STUDY00019011 (approved October 2023). All items from the World Health Organization trial registration dataset [50] are described in in Multimedia Appendix 1. UW and its IRBs follow recommendations for participant safety given by the Belmont Report and the Declaration of Helsinki [51]. Furthermore, our study consultants from STRONG STAR Training Initiative at the University of Texas Health Science Center at San Antonio consulted on assuring military cultural competency in all study activities. The principal investigator, coinvestigators, and lead research scientists monitor data and safety. In addition to the UW IRB, a data safety monitoring board (DSMB) was established, consisting of 3 research scientists with relevant experience who were not affiliated with the study, the funder, or the sponsor. All adverse events are reported to the UW IRB and DSMB per approved protocols. When relevant, any necessary amendments will be submitted to the IRB and DSMB for their approval. This trial has been registered on ClinicalTrials.gov (NCT06136234).

#### Informed Consent

The informed consent process was designed to be interactive within the REDCap (Research Electronic Data Capture; Vanderbilt University) secure online environment. To facilitate the informed consent process, participants are presented with a video that walks them through the study procedures. REDCap prevents participants from advancing to the next page for the first 3 minutes to encourage participants to engage with the video (which is just over 7 minutes long). They then proceed to the next page, where they can review the consent document in its entirety (provided in Multimedia Appendix 2). To ensure complete understanding, participants are asked 10 true or false questions about the study procedures, payment structure, and safety measures that are in place. The REDCap page allows 2 chances to answer each question correctly, and only those participants who answered correctly are asked whether they want to participate in the study. Throughout this process, participants are reminded that study staff are available by request to clarify all informed consent documents and study procedures via telephone or videoconference. They are also reminded that at any time they can (1) refuse to complete any or all the surveys and EMAs, (2) decline the qualitative interview, (3) opt out of receiving the intervention messages, or (4) withdraw from the study entirely.

#### Privacy and Confidentiality

The study team makes every effort to protect participant privacy and confidentiality. Deidentified participant data are stored in Health Insurance Portability and Accountability Act–compliant, encrypted servers provided by UW Medicine. Identifiable information is only used for the purposes of contacting participants to complete the follow-up surveys and to send the Caring Contacts intervention messages when applicable. Access to participant data is limited to certain members of the study team and any individuals from the UW or other agencies that may need to review study records, all of whom are required to complete training in human participants research and Good Clinical Practice. Participants are made aware of these protections during the informed consent process, wherein it is also explained that if we have strong reason to believe that they are in danger of suicide, we will take steps to save their life. This may involve sharing information with clinicians or emergency services in their community. If they inform us that they are going to hurt a person they identified to us, we will connect them with someone who can help prevent the danger or alert the person. In addition, we disclose that we are legally required to report to local child protective services if participants inform us that a child is at risk of abuse or neglect.

#### Compensation Details

Depending on their assigned study group, each participant may earn up to US $290, which will be paid via Amazon gift cards. The payment structure is described in the Payment Structure section in detail.

### Risk Management

Participant suicide risk is managed using the University of Washington Revised Risk Assessment Protocol (UWRRAP) [52-54]. Quantitative assessments (surveys and EMA) use the UWRRAP–online version, which is programmed to provide resources where appropriate throughout the REDCap flow that is activated if participants endorse elevated levels of SI, urges, or a recent suicide attempt for which they did not get treatment. The risk assessment or response is multitiered depending on the severity of a participant’s score, including but not limited to the following:

- On-screen reminders about the National Suicide and Crisis Lifeline, the Crisis Textline, and instructions on how to reach the veteran-specific crisis line
- In-survey prompts for participants to report how they would respond to negative feelings or suicidal thoughts and whether they have any fun or distracting activities planned for the day
- Prompts to identify safety contacts
- Crisis line information (provided by email, text, or phone, depending on patient preference), along with study contact information and hours of availability

EMA responses indicating elevated responses trigger alerts to the study staff, who review participant responses on a case-by-case basis, involving licensed psychologists on the team as needed. When appropriate, a member of the research study staff follows up with the participant and offers the needed resources, as well as crisis options. Qualitative interviews use the standard UWRRAP, which is designed for interview-based assessment.

### Eligibility Criteria

To be eligible, participants must be aged at least 18 years, speak English, be a US service member or veteran, live in the United States, be willing to be contacted by SMS text message on their personal cell phone, and endorse at least one marker of either stress or distress. Inclusion criteria for stress, or external markers, include unemployment, financial strain, housing instability, recent separation from the military, or recent sudden loss. Inclusion criteria for distress, or internal markers, include depression, substance abuse, loneliness, defeat, hopelessness, psychological pain, suicide cognitions, or the presence of SI. Each criterion’s definition and operationalization is described in the Measures section.

### Randomization

**Figure 1:**
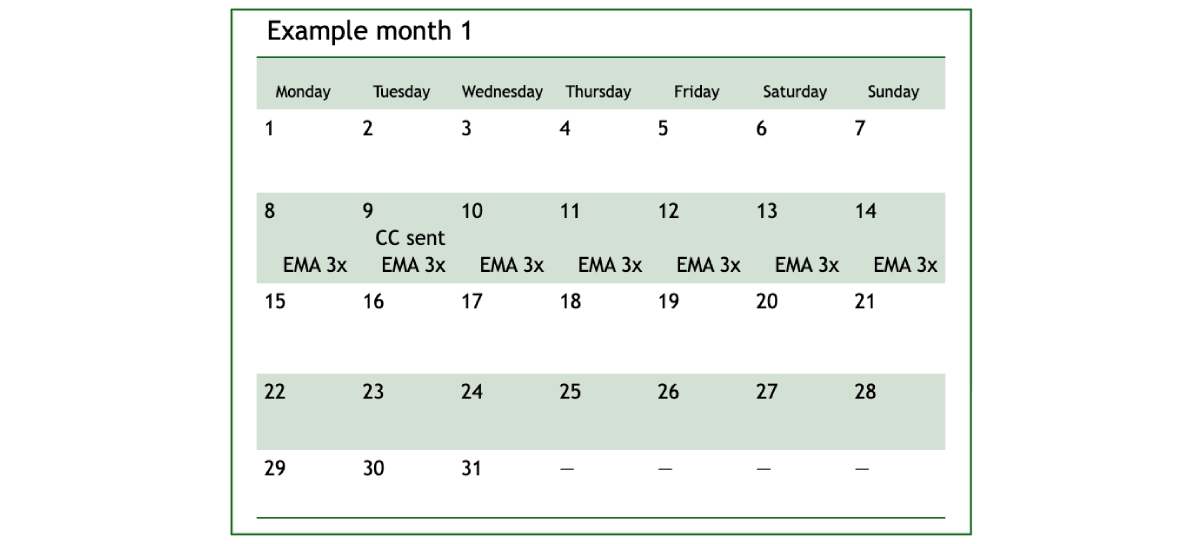
Example of an ecological momentary assessment with Caring Contacts (“EMA with CC”) month (ie, months 1, 3, 4, 7, and 12) wherein a CC message is delivered within the EMA period.

**Figure 2:**
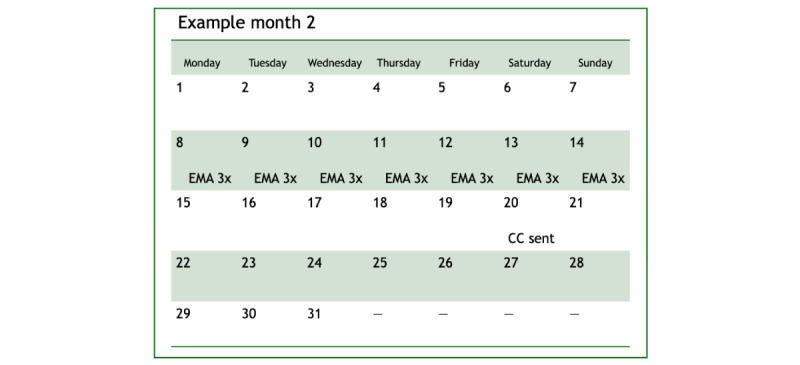
Example of an ecological momentary assessment without Caring Contacts (“EMA without CC”) month (ie, months 2, 6, 9, and 11) wherein a CC message is delivered outside the EMA period.

A randomization module with participants stratified into 2 levels (recent suicidality vs no recent suicidality) and randomized into 3 conditions (Caring Contacts with EMA, Caring Contacts alone, and EMA alone) was built into REDCap. Recent suicidality is indicated by a participant endorsing that they have either wished they were dead or had thoughts of killing themself sometime in the past year. This module is programmed to randomize participants with no involvement of study staff to prevent potential bias, with intervention and control assignments distributed evenly across participants. Once participants have completed the online baseline survey, they are randomized into 1 of the 3 conditions (Table 1; Figures 1 and 2). Further details on the EMA procedures are provided in subsequent sections.

**Table 1:** Assessment and intervention message timeline^a^.

Study procedure		Study condition		
		CC^b^ and monthly EMA	CC only	Monthly EMA only
Consent and baseline survey		✓	✓	✓
Two weeks of baseline EMA^c^		✓	✓	✓
**Randomization to study condition**				
	Month 1	EMA with CC	CC	EMA
	Month 2	EMA without CC	CC	EMA
	Month 3	EMA with CC	CC	EMA
	Month 4	EMA with CC	CC	EMA
	Month 5	EMA only	—^d^	EMA
	Month 6	EMA without CC	CC	EMA
	Month 7	EMA with CC	CC	EMA
	Month 8	EMA only	—	EMA
	Month 9	EMA without CC	CC	EMA
	Month 10	EMA only	—	EMA
	Month 11	EMA without CC	CC	EMA
	Month 12	EMA with CC	EMA with CC	EMA
Follow-up survey		✓	✓	✓
Possible qualitative interview		✓	✓	✓

^a^Three other intervention messages are delivered at various times during the study year (birthday, New Year’s holiday, and Veterans Day). Please refer to Figures 1 and 2 for examples of months that are labeled as “EMA with CC” and “EMA without CC.”

^b^CC: Caring Contacts; indicates a Caring Contacts message is sent to participants in that condition.

^c^EMA: ecological momentary assessment; indicates that 3 EMAs per day are delivered for a week to participants in that condition during that month.

^d^Not applicable.

### Payment Structure

Depending on whether they are assigned to a study group that includes EMAs throughout the year, participants may earn up to either US $180 or US $290. Reimbursement is provided via Amazon gift cards as presented in Textbox 1.

**Payment structure for the participants.**

- US $25 for the baseline survey and completing one ecological momentary assessment (EMA; ie, fully enrolling in the study) within 2 weeks
- US $2 to $10 per week of 3 EMAs each day (EMAs generally take between 30 s and 3 min to complete). For each week of surveys, payment will be as follows:
  US $2 if any EMAs completed
  US $4 if >5 but <50% of EMAs completed
  US $6 if 50% to 89% of EMAs completed
  US $10 if ≥90% of EMAs completed
- Each participant may earn a US $50 bonus if 80% of all assigned weeks of EMAs are completed (across the baseline and study year EMAs)
- US $25 for the follow-up survey (at the end of 12 and 1/2 months)
- US $50 for the qualitative interview (if they are selected)

### Enhanced Usual Care

As the study is conducted entirely online and captures participants from across the United States, where access to behavioral health and other resources for stress and distress can vary widely and is sometimes extremely limited, usual care might be inadequate, and we want to assure some benefit for all study participants. Therefore, participants in all conditions receive enhanced usual care wherein they are provided resources tailored to the participant’s needs and location. This approach is similar to the one conducted by the study team for a previous study (Hebert et al [33] and Comtois KA, unpublished data, July 2025) and as recommended by experts in treatment effectiveness studies [55,56]. Tailoring is based on each participant’s selection of the needed resources, along with the inclusion of crisis and mental health resources if suicide risk items are endorsed. Participants are asked if they want resources in the following domains: online and community resources focused on crisis and mental health support; veteran and military-specific services; relationship support; and practical assistance with food, housing, medical care, employment, education, and financial needs.

Study staff maintain a library of resources in these areas to share with participants and conduct further research when participants request other resources (examples of requests include wellness, advocacy, hobbies, outdoor activities, and therapeutic adventures). In addition, participants whose responses indicate suicide risk are provided with crisis and mental health treatment resources. To further tailor the resources to individual preferences, participants are offered a one-on-one discussion with study staff about their resource needs via a phone or Zoom (Zoom Communications, Inc) if they would like that in addition to an emailed resource list.

### Caring Contacts Intervention

Participants randomized to receive the Caring Contacts intervention will receive 13 nondemanding, caring messages over 12 months on the following schedule: a welcome message (following their baseline assessments), one message every 3 to 5 weeks for the first 21 weeks, and one message every 8 to 9 weeks for the last 31 weeks. In addition, participants will receive messages on New Year’s Day, their birthday, and Veterans Day. The delivery day and time of each message vary throughout the year to follow a more naturalistic communication pattern and avoid appearing mechanistic or spam. Table 2 provides the examples of messages.

**Table 2:** Examples of Caring Contacts messages.

Message types	Examples
General	“Hey, this is Alex just checking in. I hope you are doing okay! Feel free to let me know how things are going if it helps.”
Seasonal	“Sending you warm wishes for a bright year ahead! 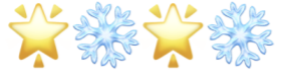 I am grateful to be in touch with you and hope this new year will be a good one, even as the seasons change – Alex”
Birthday	“Happy Birthday, Sam!! Here’s to another year 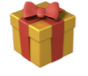 I hope you will enjoy the day!”
Veterans Day	“Happy Veteran’s Day, Sam Thank you for volunteering for service. What you’ve done is greatly appreciated. I know that this time can be difficult, if you need someone please reach out, we’re here for you if you need us. – Alex”

Caring Contacts is not designed as an automated intervention but explicitly presumes a human-in-the-loop who is choosing the messages and when they should be sent, as well as individually responding to replies from the recipients. Technology facilitates this process but does not replace it. For this study, the message schedule was determined by the lead staff, who have extensive experience with Caring Contacts, including a previous trial and our pilot work. All messages are signed with a nickname chosen by the study staff, and participants are invited (but not directly asked) to reply to the messages. The study staff respond during business hours and provide support and assistance in accessing resources as requested by the participants. The study team responds in a way that fits their personal communication style and accommodates the participant’s communication preferences, including conversation initiated by the participant. All participants are reminded that the research study team does not monitor messages outside of regular business hours and is not able to provide crisis response services.

To facilitate our assessment of Caring Contacts mechanisms, the messages are scheduled to vary across the 7-day month 1 to 12 EMA blocks, with 5 messages falling within the EMA blocks, where the impact on subsequent EMAs that day can be measured, and 5 messages outside of the EMA blocks to minimize the likelihood of participants perceiving Caring Contacts and EMA as linked. (The remaining 3 messages are holiday messages—New Year’s, Veterans Day, and their birthdays—that fall on set days and cannot easily be scheduled to fall either during or outside of the EMA periods.) Further details on the EMA procedures are found in subsequent sections.

If a participant’s reply to a Caring Contacts message indicates distress or suicidality, study staff will reply with support and validation and, as appropriate, offer crisis or other resources. If the participant is in a suicidal crisis, a “warm handoff” to 988 or another crisis line is also offered. If there is a situation involving imminent suicide risk (ie, a participant indicates they are planning a suicide attempt or have a lethal weapon) and they refuse to speak with 988 or other crisis response, research staff and licensed clinical staff or faculty consult to determine the appropriate action.

Participants are free at any time to request that they stop receiving Caring Contact messages. In such cases, study staff will inquire if they are willing to continue with the other parts of the study (EMA, the follow-up survey, and the qualitative interview), and each participant is able to decide at what level they will proceed. Participants may also ask for the messages to be paused for a certain period (eg, if they are traveling or otherwise unable to be contacted), in which case the study team will simply remove any messages scheduled to be sent during the specified period. As described in subsequent sections, the web-based platform used to deliver the Caring Contacts intervention is able to track the number of messages to and from each participant, so it will be evident during data analysis how many intervention messages each participant received. Requests to temporarily pause the messages will not change the overall length of the intervention.

We do not anticipate any reasons to withdraw participants from the intervention. However, if withdrawal is considered, the principal investigator, coinvestigators, and lead research scientists will consult together (with the UW IRB and the study DSMB as appropriate) to determine why such a decision is in the best interest of the participant and only withdraw them for that reason. In the case of a withdrawal, the principal investigator will contact the participant via email to explain the withdrawal and reasons for doing so and include an offer of a meeting to discuss further should the participant desire it.

### Caring Contacts via SMS Text Message Platform

To facilitate the scaling of Caring Contacts, our pilot work included biomedical informatics and software development methods, including human-centered design and agile prototyping, to design a web-based application to send, receive, and respond to messages with participants. Capabilities of the platform include the ability to preschedule messages and modify each message’s content and delivery date and time as needed, a conversation history display, the ability to document notes and alerts within each participant’s record for easy reference by study team members, notifications sent to specific study team members when a participant replies, and notifications to follow-up on unread messages. This platform allows oversight of all sent and received SMS text messages and the ability to export transcripts of SMS text message numbers and content with time stamps, which will be useful in analysis of the impact of Caring Contacts on participant stress and distress. Staff members had already established fidelity to Caring Contacts in our previous trial but they comonitor each other’s messages to ensure consistency with the Caring Contacts protocol for all participants.

### Study Procedures

Three different data collection procedures are used in this study.

#### Online Survey

After informed consent, participants are routed into an online REDCap survey flow to collect baseline data and route them through the steps of the study via links programmed to be sent by email or SMS text message, by the participant’s preference. At 12 months, after completion of the Caring Contacts intervention for the applicable conditions, REDCap automatically prompts the participants to complete their follow-up assessment via an online survey. The follow-up window will remain open for 3 months for each participant, and the study team will provide periodic reminders to complete the survey within the window.

#### EMA Study

Participants will receive invite links to complete the EMAs at various time points across the study period, as shown in Table 1. Each EMA point has 10 or 11 items (described in the subsequent section), which usually take 30 seconds to 3 minutes to complete (based on participants enrolled thus far and the pilot testing of the EMAs). All participants will initially receive invitations for 3 daily assessments for 14 days before randomization (42 assessments) to establish baseline mean and variability metrics for the EMA outcomes and mechanisms, and an additional 7 days of EMA (21 assessments) at 12-month follow-up. Participants randomized to conditions that include EMA, following the baseline assessment period, complete 7 days of EMA per month at months 1 to 11 (231 assessments) to assess real-time mean and variability of risk and potential mechanisms (Table 1).

#### Qualitative Interviews

A purposive subset of participants will complete a qualitative interview about their satisfaction and experience with the Caring Contacts intervention. Participants will be selected to ensure representation of gender, ethnicity, study condition, whether they responded to the Caring Contacts message, and high and low stress and distress at baseline. The interviews will continue until saturation is reached. The qualitative interview guide can be found in Multimedia Appendix 2. The data obtained during the interviews will be coded following a thematic content analysis approach, wherein a combination of deductive and inductive codes will be applied to the data, which will then be reviewed for consensus with multiple members of the research team [57,58].

### Retention Strategies

To increase retention rates, the study team uses multiple strategies. First, at the 6-month mark, the study staff reach out to participants via their preferred form of communication to confirm that their contact information is still correct and active. This check-in occurs only if (1) the participant has not responded to any Caring Contacts messages or other study-related communications, and (2) they have not completed any EMA since the end of their 2 weeks of baseline EMA.

Second, we collect a variety of contact information from each participant, including multiple phone numbers and email addresses, social media profiles, mailing addresses, and contact details of up to 2 friends or relatives who they would feel comfortable letting us reach out to if we are unable to contact them directly. This variety of contact information has been effective in keeping in touch with participants in our research, even over long stretches of time.

### Measures

Study measures are organized by primary and secondary outcomes, potential mechanisms of Caring Contacts, measures of stress, and other background measures that characterize the sample. For measures used to determine eligibility, the inclusion criterion is specified.

#### Primary Outcomes (Aims 1.1 and 1.2)

The primary EMA outcomes are mean levels of 3 suicide risk indicators of motivation to live, desire to die, and urges for suicide rated on a Likert scale from 0 to 10 (aim 1.1). The specific items include “How much do you want to die right now?,” “How much do you want to live right now?,” and “How strong are your urges for killing yourself right now?” Higher scores indicate higher suicidal risk (the item regarding motivation to live is reverse-coded). They are assessed at every EMA point throughout the study follow-up period. In the pilot trial of this study, we used visual analog scale (VAS), consisting of a slider on a straight line with end points representing the extremes, to measure these items. Their convenience and ease of rapid administration make them highly compatible with EMA research designs, facilitating efficient real-time data collection in naturalistic settings [59]. VAS of suicide intent [60], suicide desire [61], suicide urges, and perceived burdensomeness [62] were pilot-tested. However, VAS was not found to be user-friendly due to the limitations of the REDCap survey interface when presented on a smartphone. As such, we changed the response options for these suicide risk items to buttons labeled 0 through 10 in place of a VAS from 0 to 100.

The primary survey outcomes of suicide risk are administered at baseline and follow-up (aim 1.2). Suicide ideation is measured with the 12-item Suicide Ideation subscale of the Harkavy-Asnis Suicide Scale (HASS) [63] on a 5-point Likert scale, from 0 (“never”) to 4 (“most or all of the time”). Higher scores indicate higher SI. While the original scale assesses for the past 2 weeks, this trial uses the measure to assess for the past month as recommended by the measure’s creators, and rather than 4 indicating “daily,” it indicates “most or all of the time.” An example question from this version of the measure is “Over the past month, how often have you...” with response options including the following: “Thought you would be better off dead?,” “Had ideas about killing yourself?,” and “Thought about killing yourself but did not try to do it?” This modified version of the HASS self-report measure has been validated by Asarnow et al [64]. The measure has also been demonstrated to have strong psychometric properties in youth samples, with good internal validity and high sensitivity to treatment changes [65]. The eligibility criterion for SI is a score of 3 or higher. The 6-item Brief Suicide Cognitions Scale [66], a shortened version of the Suicide Cognitions Scale-Revised [67], is also used to assess suicide cognitions without asking about SI or attempts and is administered before the Suicide Ideation subscale of the HASS. Items are scored on a 5-point Likert scale from 1 (“strongly disagree”) to 5 (“strongly agree”). Higher scores indicate more suicidal cognition. The Suicide Cognitions Scale-Revised has been validated with a sample of military adults by Fischer et al [68], and the Brief Suicide Cognitions Scale was used successfully with a sample of US military veterans [67]. The eligibility criterion for suicide cognitions is a score of at least 4 on any item.

#### Qualitative Interviews (Aim 2)

The qualitative interview—modeled on similar interviews in previous Caring Contacts research [33]—asks participants about their experience with the Caring Contacts intervention (including receiving and replying to the messages and responses they received from the author, and satisfaction with and preferences for the intervention messages), as well as their experience completing the EMAs. The interview content was refined during the pilot test of this trial with military veterans and clinical participants (manuscript currently in preparation). The qualitative interview guide is provided in Multimedia Appendix 3.

#### Distress: Secondary Outcomes (Aim 3)

Depression is assessed with the Patient Health Questionnaire-9 [69], a standard and widely used brief measure of depression severity. A total of 9 items are scored on a 4-point Likert scale from 0 (“not at all”) to 3 (“nearly every day”). Higher scores indicate a higher degree of depression severity. An example question from this version of the measure is “Over the last 2 weeks, how often have you been bothered by any of the following problems?” with response options including the following: “Little interest or pleasure in doing things” and “Feeling tired or having little energy.” The measure has been validated as a useful tool to assess depression in clinical and general populations [69,70]. The eligibility criterion for depression is a score of 10 or more.

Substance use issues are measured by the Short Inventory of Problems (SIP)-Alcohol and Drugs [71], a 15-item psychometrically sound measure [72] assessing the frequency over the past 2 weeks of adverse consequences of alcohol and drug use in 5 domains: interpersonal, intrapersonal, physical, impulse control, and social [73]. Items are scored on a 4-point Likert scale from 0 (“never”) to 3 (“daily or almost daily”), where higher scores indicate increased severity of drug and alcohol problems. An example question from this version of the measure is “During the past 2 weeks, about how often has this happened to you?” with response options including the following: “I have been unhappy because of my drinking/drug use,” and “When drinking/using drugs, I have done impulsive things that I regretted later.” The version of this measure, the SIP-2L, which is worded specifically to assess drinking problems, demonstrated strong internal consistency among items, and correlations with self-reported heavy drinking demonstrated convergent validity in a sample of adults with alcohol use disorder recruited for a harm-reduction trial [72,74]. A similar version of the SIP-Alcohol and Drugs, the SIP-Revised, has been shown to have excellent internal reliability and convergent validity in measuring substance use consequences in a sample of treatment-seeking drug users [73] The eligibility criterion for substance use issues is a total score of 3 or more, or any singular item rated at least 2.

Loneliness is assessed with the National Institutes of Health (NIH) Toolbox Loneliness Scale from the Adult Social Relationship Battery [75]. Participants are asked to rate how often in the past month they have, “felt alone and apart from others,” “felt left out,” “felt lonely,” etc on a 5-point Likert scale of 1 (“never”) to 5 (“always”). Higher scores indicate more loneliness. The scale was demonstrated to have convergent validity with 3 other validation instruments [75]. The eligibility criterion for loneliness is a total score of 15 or higher, or any 2 items scored at least 5.

A sense of defeat is measured using the 16-item Defeat Scale [76,77]. Items are scored on a 5-point Likert scale from 0 (“never”) to 4 (“always”) where higher scores indicate greater feelings of defeat. Items include, “I feel that I have not made it in life,” “I feel I have lost important battles in life,” and “I feel that there is no fight left in me.” Three of the items are reverse-coded and include, “I feel that I am a successful person” and “I feel able to deal with whatever life throws at me.” The scale was shown to have high levels of internal consistency and to be psychometrically sound by the authors [76]. The eligibility criterion for defeat is a score of 24 or more.

Feelings of hopelessness are measured using the Beck Hopelessness Scale (BHS) [78], which is composed of 20 true or false items (9 of which are reverse-coded). While the psychometric properties of the BHS have not been tested in US military samples, the scale has demonstrated good convergent validity across various populations. The scale has been shown [79-81] to be internally consistent with strong concurrent validity with other psychiatric measures, albeit in primarily clinical settings. It significantly correlates with measures of depression, anxiety, and SI [82-84] and has been validated in both clinical and nonclinical populations, including university students, general population samples, and caregivers of patients with psychiatric disorders. In addition, the BHS is sensitive to changes in depression over time [84-86]. The eligibility criterion for hopelessness is a score of 9 or more.

The extent to which an individual is experiencing psychological pain is measured using the 3-item Unbearable Psychache Scale [87]. Items are scored on a 5-point Likert scale from 1 (“strongly disagree”) to 5 (“strongly agree”). Higher scores indicate that one’s psychological pain is more unbearable. An example item is “The following statements refer to your psychological pain, not your physical pain. Please indicate the extent to which you disagree or agree with each” with response options including the following: “My pain is making me fall apart” and “Because of my pain, my situation is impossible.” This brief measure was demonstrated to be a valid and reliable measure of psychache [88]. The eligibility criterion for psychache is a score of 9 or more.

#### Potential Mechanisms of Caring Contacts (Aim 4)

To evaluate how Caring Contacts influence connectedness, we selected 4 mechanisms for testing based on prominent psychological suicide theories [40,89,90]: burdensomeness, entrapment, mattering, and personal and social responsibility. Measures of these possible mechanisms of action for the Caring Contacts intervention are administered through EMA and the baseline and follow-up surveys.

Two of the potential mechanisms, perceived burdensomeness and entrapment, are each assessed with a single question selected based on prior EMA studies studying suicide.

Perceived burdensomeness has consistently emerged as a robust predictor of SI, reflecting the harmful impact of feeling like a burden to others. During the EMA period, it is assessed using a slightly modified version of an item from the Interpersonal Needs Questionnaire [91] (“I felt that I was a burden to other people, or that they would be better off without me”), which has been used in previous EMA research [92].

Entrapment is a key construct in the integrated motivational-volitional model [40] of suicide and represents the psychological state of feeling trapped with no escape, which is strongly associated with suicidal intent. In this study, it is assessed at baseline and follow-up using the 16-item Entrapment Scale [76,77]. Items are scored on a 5-point Likert scale from 0 (“not at all like me”) to 4 (“extremely like me”) and include “I am in a situation I feel trapped in” and “I would like to get away from who I am and start again.” During the EMA period, it is assessed using a single item from the scale (“I feel trapped”). Higher scores indicate increased feelings of being trapped. The measure’s authors found it to be basically unidimensional and psychometrically valid [77].

A planned missingness design was implemented to reduce the length of each individual EMA, thereby lowering the effort needed by participants to complete it. Thus, 3 of the mechanisms that are measured with multiple items—mattering, personal responsibility, and social responsibility—are assessed in a random rotation at each of the 3 EMA points within a day.

Mattering is how an individual perceives their significance and importance to others, which directly counters feelings of insignificance and isolation. In this study, it is measured using the 5-item General Mattering Scale [93-96] at baseline, follow-up, and in the EMA. Items are scored on a 4-point Likert scale from 1 (“not at all”) to 4 (“a lot”) and include “How much do other people depend on you?” and “How important do you feel you are to other people?” Higher scores indicate increased feelings of mattering to others. In contrast to mattering, antimattering is the extent to which an individual feels insignificant or worthless. It is valuable to assess both mattering and antimattering as it has been postulated that these constructs are not opposites of each other but rather capture distinct protective and risk factors [97-99]. The 5-item Anti-Mattering Scale is administered with the General Mattering Scale at baseline and follow-up. Items are scored on a 4-point Likert scale from 1 (“not at all”) to 4 (“a lot”) and include “How much do you feel like you don’t matter?” and “To what extent have you been made to feel like you are invisible?” Higher scores indicate greater feelings of antimattering.

In this trial, personal and social responsibility are defined as an individual’s feeling of accountability toward their own well-being and the well-being of others. They speak to an individual’s sense of belonging within broader social systems and reinforce protective roles within their network. In this study, they are measured by the two 4-item subscales of the 8-item Responsibility Scale [100] at baseline, follow-up, and during the EMA period. Items are scored on a 5-point Likert scale from 1 (“strongly disagree”) to 5 (“strongly agree”). Items from the personal responsibility scale include “I discipline myself to make the best use of my time doing meaningful things” and “I am conscientious in whatever I do, big or small.” Items from the social responsibility scale include “I am morally accountable for how I treat others” and “I am responsible to do my part to make the world a better place.” Higher scores indicate a greater sense of personal and social responsibility [100].

#### Measures of Stress

Protocols from the NIH-funded PhenX Toolkit’s Social Determinants of Health Core Collection [101] are used to assess external stress, including current employment status (unemployed, looking for work, or disabled); food insecurity; access to health services; and housing instability due to affordability. Recent military separation was assessed as separation within the past year. For consistency, we have adopted these protocols for our demographic and health disparities measures. However, many items do not have previous literature in the survey form and therefore had to be adapted for this study. The eligibility criteria for each of these stressors are provided in Textbox 2.

**The eligibility criteria for the measures of stress.**

- Unemployment: participants indicate that they are looking for work, unemployed due to being temporarily laid off, on sick leave, or on parental leave, or temporarily or permanently disabled.
- Financial strain: participants indicate that they are unable to afford adequate food or health care service.
- Unstable housing: participants indicate that they are living in a temporary or unreliable housing situation, or that they have been worried about being able to afford a place to live for most of the last 12 months.
- Recent military separation: participants indicate that they left the military (including Reserves and National Guard) in the last 12 months.

A modified version of Cerel’s Exposure to Loss measure [102,103] is used to assess the number of sudden losses, both suicide-related and nonsuicide-related, that participants have experienced, as well as he timing of the most recent loss, the extent to which the participant continues to feel impacted by the loss, and their relationship with the person who had died. Closeness is measured with a single item on a 5-point Likert scale from 1 (“not close”) to 5 (“very close”). Impact of the loss is measured with a single item on a 5-point Likert scale from 1 (“The death had little effect on my life”) to 5 (“The death had a significant or devastating effect on me that I still feel.”). These items have been used successfully to capture participants’ self-reported impact of their losses in other studies with both clinical [104] and military veteran samples [105] and are recommended to assess for psychological distress due to loss. The eligibility criterion for recent sudden loss includes experiencing either a suicide or nonsuicide death that occurred within the last year and had a moderate effect on their life, or occurred more than a year ago but had a significant or devastating impact that the participant still feels.

#### Other Measures That Characterize the Sample

The Columbia-Suicide Severity Rating Scale (C-SSRS) screener [106-113] assesses recent suicidal behavior, including preparatory behavior, and aborted, interrupted, and actual attempts. To maximize readability, the pediatric or cognitively impaired C-SSRS version [114] was selected for this study, and additional items were developed and added to gather additional details on suicidal behavior. The C-SSRS screener has strong psychometric properties demonstrated in general [113] and veteran [108] populations, is used in the Defense Health Agency’s Behavioral Health Data Portal [115]**,** and has shown predictive validity for suicidal behavior. The eligibility criterion for recent SI is having SI in the past month or making an aborted, interrupted, or actual suicide attempt in the last month.

Participants report their usual sleep and wake schedules at baseline, follow-up, and on the last day of each week of EMA. Items from the Consensus Sleep Diary [116] and the Patient-Reported Outcomes Measurement Information System (PROMIS) Sleep Disturbance Scale [117,118] ask about recent sleep quantity and quality. The Consensus Sleep Diary was shown to be a valid and reliable measure of sleep in multiple studies [119-121] while the PROMIS Sleep Disturbance scale was shown to have good face and construct validity, convergent validity, and internal consistency reliability [118].

Various protocols from the NIH-funded PhenX Toolkit’s Social Determinants of Health Core Collection [101] are used to assess demographic characteristics, such as age, gender identity, sexual orientation, race, ethnicity, living situation, occupation, and highest education received. They are supplemented with military-specific questions, such as branch, rank achieved, military occupation, deployments, and services in Reserves or National Guard.

### Data Analysis

#### Power and Sample Size

We plan to recruit 510 participants with the aim of holding type I (α=.05) and type II error rates (β=.05; power=0.95) at equal levels. For aim 1.1 with 3 conditions and 2 assessment time points, a sensitivity power analysis conducted using the *pwr* package in the R software (R Foundation for Statistical Computing) indicated that our proposed sample size will provide sufficient power to detect moderate effect sizes, with the smallest detectable effect size being f^2^=0.17. For aim 1.2, our proposed sample size will provide sufficient power to detect small effect sizes, with the smallest detectable effect size being f^2^=0.03. On the basis of the prevalence rate for SI in US military service members and veterans between 13.9% and 14.1% and between 1.9% and 2.4% for suicide attempts [122], we expect that 13.9% (71/510) of our proposed full sample will report current SI.

For aim 3, comparing the 2 primary conditions (n=340), with a 7-day assessment period and 3 assessments per day (total observations, n=21), power analysis using the *EMAtools* R package shows sufficient power (0.95) to detect moderate (Cohen *d=*0.5) to large (Cohen *d*=0.8) effects within a month at α=.05, even with a 50% response completion rate. For the full year of the monthly 7-day assessment period (total observations, n=252), the same analysis similarly indicates sufficient power to detect moderate to large effects across 12 months. For aim 4, the recommended sample size is for a 2-level mediation model based on Monte Carlo simulations [123] or detecting small effects with adequate power (0.8) while holding the type I error rate at α=.05, when there are at least 3 assessments per participant, are between 215 and 257 participants if within-participant correlations are moderate (intraclass correlation coefficient [ICC]=0.1),300 to 438 participants if within-participant correlations are moderate (ICC=0.4-0.6), and 495 to 544 participant if within-participant correlations are high (ICC=0.9). Notably, as within-participant observations increase, the required sample size to achieve adequate power decreases. With 12 within-participant observations, our required sample size would be smaller than the recommended sample size [123]. Altogether, 510 participants and 252 assessments per participant over the course of the study should provide sufficient power to detect the hypothesized between- and within-participant effects.

#### Analysis Plan

First, descriptive statistics will be calculated for all variables and group differences in baseline variables will be examined to ensure randomization success. All outcome variables will be examined for nonnormality, and given past suicide research, we anticipate a positive skew for suicidal thoughts and behaviors, and we will use an appropriate model for all analyses based on the distribution of the outcome variables. On the basis of a Directed Acyclic Graph model generated using DAGitty [124,125], demographic variables are conditionally independent of our outcome variables after accounting for the variables specified in our aims and thus do not confound the relationship between the Caring Contacts intervention and our outcome variables. However, to understand the nuanced effects of Caring Contacts on suicide risk across diverse subgroups, we will control for ethnicity, gender, and military status (veteran vs current US military service member) as covariates in our analysis. This stratification allows us to discern potentially differential impacts of the intervention and ensures that our findings are robust and applicable across the varying demographic profiles within the veteran population. Furthermore, all analyses will be conducted using an intent-to-treat framework, using all available data, and using robust maximum likelihood estimation to account for missing data.

For aim 1.1, individual participant means and SDs of each suicide indicator (motivation to live, passive ideation, active ideation, suicide intent, and urges to harm self) will be calculated from the baseline and 12-month EMA period (for all conditions). Then, to assess group differences in the outcome measures of interest, an analysis of covariance examining condition-specific differences at 12 months, controlling for baseline values of the outcome variable of interest, will be conducted. Preplanned post hoc comparisons will compare (1) any Caring Contacts (combined) to no Caring Contacts, (2) Caring Contacts with and without EMA, and (3) EMA with and without Caring Contacts.

For those participants who will complete EMA for all 12 months (n=340), we will conduct an *exploratory analysis* of changes over time in the mean and intraindividual SD of suicide outcomes. The unique and intensive sampling approach of EMA allows for fine-grained exploration of if and when outcomes change in a clinical trial. Specifically, we will first visually examine trajectories by condition to determine the shape of change *trajectories* over time. Then, following the determination of the shape, we will conduct a linear mixed effects growth model (eg, linear, nonlinear quadratic, and piecewise) [126-129], including a dummy-coded condition variable (with EMA only as the reference) as a predictor of both intercept and slope to determine how intervention condition impacts change in suicide outcomes over time.

For aims 1.2 and 3, to examine secondary suicide outcomes and measures of psychological distress, we will use analysis of covariance to examine condition-specific differences in continuous scores of suicide ideation and cognitions and examine group differences at 12 months, controlling for baseline values of the outcome variable of interest. Similarly, preplanned post hoc comparisons will compare (1) any Caring Contacts (combined) to no Caring Contacts, (2) Caring Contacts with and without EMA, and (3) EMA with and without Caring Contacts. For both aims 1.1 and 1.2, any baseline variables that were not successfully randomized will be included as covariates in the regression models.

For aim 2, thematic analysis will be used to systematically examine the qualitative data obtained from the interviews [57,58]. This process will begin with verbatim transcription of the interview audio recordings, ensuring a precise and comprehensive dataset. An inductive approach will be adopted, allowing themes to emerge organically from the data without preconceived categories. The research team will use open coding, assigning descriptive codes to individual text segments within each transcript. This initial coding will facilitate the identification of patterns and key themes related to participants’ experiences and perceptions of the Caring Contacts intervention. To ensure accuracy and consistency, intercoder reliability will be established through regular team meetings where coding decisions are discussed and consensus is reached. This iterative process will continue as new codes emerge, with the team members actively refining and redefining codes to capture the nuances of the data accurately. Following open coding, the research team will engage in axial coding, grouping codes into broader categories to elucidate connections and relationships between them. This step lays the foundation for developing overarching themes that encapsulate the study’s core insights. Finally, the coded data will be systematically reviewed and organized to refine these themes and develop a coherent narrative that accurately reflects the participants’ experiences. Triangulation methods will be used throughout this process, comparing information from different participant groups and cross-referencing existing literature to validate the findings and ensure their robustness and reliability.

For aim 4, to examine potential mechanisms of Caring Contacts, we will use the within-EMA period data and examine within-participants mediation using dynamic structural equation modeling (with random intercepts and slopes) [130]. Dynamic structural equation modeling allows for the simultaneous estimation of within- and between-person effects that allow for idiographic mechanistic models over time, which can be ideal for answering questions about within-participant mechanisms of suicide outcomes. In line with recommendations for intensive longitudinal data analysis, all hypothesized mechanism variables (ie, mattering, personal and social responsibility, perceived burdensomeness, and entrapment) will be centered at both the between-person and within-person mean level. Then, within-person regression models will be run to examine the relationship between the hypothesized mechanisms and suicide outcome, using a lag-1 model and autoregressive covariance structure to account for assessments closer in time to be more highly correlated. Next, to examine the mediation effect of treatment condition on outcomes through the hypothesized mechanisms, we will estimate within-level indirect mediation effects, calculated as the product of the within-level effect of treatment condition on the mechanism (a path) and the effect of the mechanism on the suicide outcome. All mediation analyses will be examined at the within-person level (1-1-1), excluding cross-level mediation. Random starts will be used for all models to replicate the best log-likelihood. Finally, to examine the immediate versus delayed impact of the timing of Caring Contacts intervention, we will use autoregressive modeling to examine the direct impact on stress, distress, and suicide outcomes immediately following a Caring Contacts message compared to times without a message, using the EMA data. For all regressions, 95% CIs will be reported, and statistical significance will be set at *P*<.05.

## Results

Recruitment for this RCT started in April 2024 and is projected to close in May 2025, or until the target sample size of 510 participants is reached. As of December 2024, we have enrolled 321 participants. Final quantitative data collection is projected to be completed by July 2026, when the final 12-month follow-up windows close. Data from qualitative interviews will be collected until September 2026. Data analysis will occur following the data collection, and the results are expected to be published in winter 2027.

## Discussion

### Significance

This innovative trial will be the first RCT to examine (1) the impact of the Caring Contacts intervention on a broad population experiencing stress or distress and (2) the mechanisms of Caring Contacts, both when each message is received and during the course of the 1-year intervention. By recruiting veterans and service members experiencing a wide variety of stressors and psychological distress, the sample is more generalizable to those served by veteran service organizations, primary care, and general behavioral health care. If Caring Contacts reduces the risk of suicide, improves psychological distress, or both, then the intervention could be scaled up to include individuals who do not endorse suicidality but have the potential to benefit. As veteran service organizations are not equipped to systematically assess suicidality or to provide an intervention specifically for this segment of their membership, positive results would support their implementation with any veteran experiencing stress or distress.

EMA provides a powerful tool for measuring the impact of Caring Contacts by enabling real-time data collection in participants’ natural environments. This approach minimizes recall bias and allows a detailed exploration of how the intervention affects participants immediately after receiving Caring Contacts and over the course of 1 year. Thus, EMA allows researchers to gain valuable insights into the mechanisms of action of the Caring Contacts intervention. This can address critical gaps in understanding and pave the way for improved, scalable strategies to prevent suicide.

### Limitations

For participants randomized to receive both Caring Contacts and EMA, the effect size of Caring Contacts may be diluted due to the increased contact associated with EMA. This is mitigated by including 2 other conditions—Caring Contacts without EMA and EMA without Caring Contacts—and by the EMA and Caring Contacts coming from different phone numbers, thus appearing as separate conversation threads on participants’ cell phones. Another limitation is the risk of a smaller effect size across all study conditions from the enhancement of usual care with the provision of tailored resources to each participant. Given that all the veterans and service members were experiencing stress or distress and are at outsized risk of suicide, the risk that they did not have the resources they needed or the knowledge and access to find them meant that treatment as usual was ethically inadequate and offering what could be offered was important. Online research is inherently limited by the challenge of verifying the authenticity of participants. In this trial, we offset this risk with multiple automated and human-in-the-loop checks to prevent fraudulent participation. Finally, we are using EMA and survey assessments of suicidal thoughts and behaviors that are subject to the limitations inherent in self-report measures. However, EMA measures were selected based on previous research, standardized scales were used for survey assessments, and randomization will equate any self-report bias across the 3 conditions.

### Conclusions

To conclude, this innovative RCT is designed to evaluate the Caring Contacts intervention for veterans and service members experiencing stress or distress while simultaneously evaluating potential mechanisms through which this evidence-based intervention is working. The EMAs right before and after Caring Contacts messages are received, as well as the monthly EMA tracking changes in mechanisms and outcomes during the course of the 1-year intervention, offer one view, while qualitative interviews with study participants offer another view into the impact of Caring Contacts. This is also one of the first trials for suicide prevention that focuses on EMA as the primary outcome. Results from this project will further inform optimal implementation of Caring Contacts and provide crucial insights to address the urgent need for practical, scalable mental health solutions.

### Disclaimer

The views expressed herein are those of the authors and do not necessarily reflect the official policy of the sponsor or the funder.

## Appendix

Multimedia Appendix 1 World Health Organization trial registration dataset.

Multimedia Appendix 2 Example consent form.

Multimedia Appendix 3 Qualitative interview guide.

## Abbreviations

BHS: Beck Hopelessness Scale
C-SSRS: Columbia-Suicide Severity Rating Scale
DSMB: data safety monitoring board
EMA: ecological momentary assessment
HASS: Harkavy-Asnis Suicide Scale
ICC: intraclass correlation coefficient
IRB: Institutional Review Board
NIH: National Institutes of Health
PROMIS: Patient-Reported Outcomes Measurement Information System
RCT: randomized controlled trial
REDCap: Research Electronic Data Capture
SI: suicidal ideation
SIP: Short Inventory of Problems
UW: University of Washington
UWRRAP: University of Washington Revised Risk Assessment Protocol
VAS: visual analog scale

## Data Availability

The datasets generated or analyzed during this study will be available from the corresponding author on reasonable request.
