# Supplementary material for: Exploring the Impact of the Caring Contacts Intervention on the Stress and Distress of Veterans and Service Members: Protocol for a Randomized Controlled Trial
Source: JMIR Res Protoc. 2025 Aug 13;14:e72140. doi: 10.2196/72140 (PMC12391844; doi:10.2196/72140)
Supplement: Multimedia Appendix 1 [file resprot_v14i1e72140_app1.docx]

**Supplemental Table 1**. Items from the World Health Organization (WHO) trial registration dataset.

| Data category | Information |
| --- | --- |
| Primary registry and trial identifying number | - ClinicalTrials.gov NCT06136234 |
| Date of registration in the primary registry | - 18 November, 2023 |
| Secondary identifying numbers | - University of Washington IRB: STUDY00019011 |
| Sources of monetary or material support | *Face the Fight Charitable Fund* |
| Primary sponsor | - University of Washington |
| Contact for public queries | - BW, AE, KAC, [uwcspar@uw.edu](mailto:uwcspar@uw.edu) |
| Contact for scientific queries | - BW, AE, KAC, [uwcspar@uw.edu](mailto:uwcspar@uw.edu) - University of Washington |
| Public title | - Caring for Vets and Service Members |
| Scientific title | - Caring Contacts for Stressed and Distressed Veterans and Service Members |
| Countries of recruitment | - United States |
| Health conditions or problems studied | - Stress, emotional distress |
| Interventions | - Caring Contacts (message-based suicide prevention intervention) - Enhanced usual care |
| Key inclusion and exclusion criteria | - Ages eligible for study: ≥18 y - Sexes eligible for study: both - Accepts healthy volunteers: no - Inclusion criteria: US military service member or veteran; ≥18 y; lives in the United States, stressed (recent separation from or in transition out of the military, unemployment, financial strain, unhoused, or suicide loss, etc) or distressed (isolation, depression, substance use, loneliness, defeat, hopelessness, psychological pain, or suicidal ideation, etc); willing to be contacted periodically by text message - Exclusion criteria: unable to consent due to inability to understand the consent form due to cognitive limitations or insufficient English (as determined by inability to pass the consent quiz items) |
| Study type | - Interventional - Allocation: randomized intervention model. Parallel assignment with 3 arms. Masking: primary outcomes all collected via self-report - Primary purpose: treatment - Phase: not applicable |
| Date of first enrollment | - April 2024 |
| Target sample size (n) | - 510 |
| Recruitment status | - Recruiting |
| Primary outcomes | - Suicide risk indicators (motivation to live, passive ideation, active ideation, and urges for suicide) rated on Visual Analog Scales acquired during EMA^a^ periods, administered at 3 EMAs per day for 14 days at baseline; then either 3 EMAs per day for 7 days at 12 months or 3 EMAs per day for 7 days per month for 12 months (depending on EMA condition) - Suicidal ideation via Harkavy-Asnis Suicide Scale (adapted version for study procedures), administered at baseline and 12-month follow-up - Suicide cognitions via Suicide Cognitions Scale - Revised, administered at baseline and 12-month follow-up |
| Key secondary outcomes | - Experience of receiving Caring Contacts, administered at 12-month follow-up - Depression via Patient Health Questionnaire-9 administered at baseline and 12-month follow-up - Substance abuse via Short Inventory of Problems—Alcohol and Drugs, administered at baseline and 12-month follow-up - Loneliness via National Institutes of Health (NIH) Loneliness Scale, administered at baseline and 12-month follow-up - Defeat via the Defeat Scale, administered at baseline and 12-month follow-up - Hopelessness via Beck Hopelessness Scale, administered at baseline and 12-month follow-up - Psychological pain via Unbearable Psychache Scale-3, administered at baseline and 12-month follow-up |

^a^Ecological momentary assessment.
