# Supplementary material for: Exploring the Impact of the Caring Contacts Intervention on the Stress and Distress of Veterans and Service Members: Protocol for a Randomized Controlled Trial
Source: JMIR Res Protoc. 2025 Aug 13;14:e72140. doi: 10.2196/72140 (PMC12391844; doi:10.2196/72140)
Supplement: Multimedia Appendix 2 [file resprot_v14i1e72140_app2.docx]

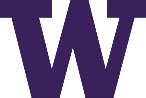
INFORMATION ABOUT A UNIVERSITY OF WASHINGTON RESEARCH STUDY

**COVE** **(Caring for Vets and Military Members)**

# What is this study about?

USAA’s Face the Fight^TM^ coalition is partnering with the University of Washington to explore how receiving messages of support might help Veterans and active-duty service members who are experiencing stress or distress.

# What will you be asked to do?

If you decide to be in this study, we will ask you to complete a series of online surveys and an interview over the span of 12.5 months, detailed below:

| **Study procedure** | **Schedule** | **Estimated time** |
| --- | --- | --- |
| **Screening for eligibility** | After completing this consent process | 15-20 mins |
| **1. Baseline survey** | Immediately following the screening | 30-45 mins |
| **2. Study Year** | Everyone will complete quick check-ins 3 times a day, for the next 2 weeks after the baseline survey. Following that, the next 12 months depends on what study group you are assigned to. | 3-5 mins each |
| Groups 1 and 2 | You will complete quick check-ins 3 times a day for 1 week, occurring once a month for the following 12 months. | 3-5 mins each |
| Group 3 | You will only complete one more week of quick check-ins during month 12 of your participation. We will send you reminders! | 3-5 mins each |
| **3. Follow-up survey** | 12.5 months from when you sign up | 30-45 mins |
| **4. Ending interview (see below for details)** | If selected | 30-60 mins |

# Why might you want, or not want, to participate?

| **Reasons you might say “yes” to being in the study.** | **Reasons you might say “no” to being in the study.** |
| --- | --- |
| - You want to help advance our understanding of how to reduce stress and distress, especially for Veterans and service members. - You’d like to complete online surveys and potentially be interviewed by study staff on your experiences of stress and distress now and over the next twelve months. - You’d like to learn about resources that might be of help to you and receive a resource list. - You are willing to receive text messages of support from study staff over the next 12 months. | - You feel uncomfortable or upset being asked personal questions regarding your stress, distress, suicidal thoughts and behavior, substance use and mental health, military history and personal characteristics. - You don’t want to complete brief surveys 3x/day for two weeks now and then possibly for a week every month over the next 12 months. - You do not want to be randomly assigned (like rolling dice) either to receive text messages or not over the next twelve months. |

# More info regarding your participation:

In addition to the online surveys, we’ll ask you to provide contact information for us to keep in touch with you over the course of the study (text, email, social media, or postal mail).

**You may refuse to answer any survey or interview question.**

**The study cannot provide mental health treatment or specific referrals to mental health treatment.** Resources will be offered to you at the end of the survey and interview. If your responses suggest you might need immediate help, assistance from a crisis support line will be offered to you. At any time in the study, you can reach out to us and the study team can provide additional resources.

## Study Groups

**We will assign you to one of three groups.** The group you are assigned to will be random, like a dice roll.

- **Group 1:** You will receive BOTH **Caring Contacts** and invites to complete 12 months of **quick check-ins**. The maximum amount you might earn is $290.
- **Group 2:** You will receive invites to complete 12 months of **quick check-ins**. The maximum amount you might earn is $290.
- **Group 3:** You will receive **Caring Contacts**. The maximum amount you might earn is $180.

| **What are Caring Contacts?**  You’ll receive caring and supportive text messages at various times. If you reply to the messages, you will get a response, but you don’t have to respond. | **What are the Quick check-ins?**  You’ll receive invitations to complete brief surveys. They should only take about 3-5 minutes each. |
| --- | --- |

**Each participant will be offered:**

- resources for the areas of stress or distress they report experiencing,
- the opportunity to meet with a member of the study team to develop a more tailored resource list, if wanted
- details about how to reach the study team,
- and details about who to reach out to if in need of immediate help.

**Some participants will be asked for an interview to discuss their experience with the messages and/or the surveys.**

- We will select participants to be sure that people with different identities are included (for example: military experience, types of stress and distress, gender, race/ethnicity).
- A researcher will ask questions about your experience with the study procedures and what you might suggest we change to make them more helpful to you.
- The interview will held on Zoom or over the phone and will last 30-60 minutes, depending on how much you’d like to share.
- With your permission, we will record the audio of this interview for future reference and research use. Consent for audio recording will be confirmed prior to the interview beginning by the research study team member who will interview you However, you will be free to decline the recording if you prefer not to be recorded while still participating fully in the interview. To further protect your privacy and the privacy of others near you, we suggest that you find a private space away from other people for the duration of the interview.

## Alternatives to Study Participation

**There are other opportunities and resources for Veterans and active duty service members who may be feeling stressed or distressed.** Please visit <https://wefacethefight.org> for more information.

## Payment

**As noted above, this is a paid study.**

You will be reimbursed via Amazon gift cards as follows:

- $25 baseline survey, enrolling in quick check-ins, and completing one check-in (i.e., fully enrolling in the study) within 2 weeks
- $2-10/week of Quick check-ins where you will receive prompts for 3 brief surveys/day or 21 surveys/week.
  - For each week of surveys, payment will be:
    - $2 if any Quick check-ins completed
    - $4 if more than 5 but less than 50% of Quick check-ins completed
    - $6 if 50-89% of Quick check-ins completed
    - $10 if 90% or more of Quick check-ins completed
  - $50 bonus if 80% of all assigned weeks of Quick check-ins are completed (across baseline and study year)
- $25 Follow-up survey (at end of 12 & 1/2 months)
- $50 for an Ending interview (if selected)

**There is no cost to you for participating.** You will be texted links to the quick check-ins throughout the study period, and you may be texted messages of care and support. Standard text messaging rates apply to those messages. You can notify the study staff if you would like your Quick check-in invitations and caring messages to be delivered via email to prevent this cost.

Because online research is vulnerable to fraud, multiple checks are employed to reduce fraudulent enrollment. Research staff will verify your submission before payment is issued. Fraudulent participation, including duplicate submissions, will be discontinued without compensation.

# How will we protect the information you provide?

**We will protect your confidentiality.** We will store your name and other identifiable information separate from the study data. Access to your identifying information will be limited to certain members of the study team and any individuals from the UW or other agencies that may need to review study records. When we publish the results of this study, we will not use your name. All information you provide is confidential. However, if we have strong reason to believe that you are in danger of suicide, we will take steps to save your life. This may involve sharing information with clinicians or emergency services in your community. If you tell us that you are going to hurt a person you identified to us, we will connect you with someone who can help prevent the danger or alert the person. If you inform us that a child is in danger of abuse or neglect, we are required by law to report the danger to the local child protective services.

**The information that we obtain from you for this study might be used for future studies.** We may remove anything that might identify you from the information. If we do so, the information may then be used for future research studies or given to another investigator without getting additional permission from you. It is also possible that in the future we may want to use or share study information/samples that might identify you. If we do, a review board will decide if we need to get additional permission from you.

# What if you want to stop being in this study?

You are free to decline further participation and withdraw from this study at any time without penalty or loss of benefits to which you are otherwise entitled. If you wish to withdraw, please contact the researcher listed at the end of this consent form. You can decline to complete any of the quick check-ins but still complete the follow-up survey, or vice versa. You can also decline to receive messages of care and support but still complete the quick check-ins and the follow-up survey, or vice versa.

# Other Information

**Being in this study is voluntary.** This means that you can refuse to sign up. It also means that if you do sign up, you can decide to stop being in the study at any time without penalty.

**We are receiving financial support** from the USAA Foundation’s Face the Fight^TM^ Coalition.

**There will be 510 participants recruited into this study.**

**The research team will also review records with the National Death Index to determine overall mortality rates of participants enrolled in the study.** National Death Index records review will occur approximately one year from your enrollment into the study with possible additional reviews over the 10 years after your enrollment.

**A PDF copy of the consent form will be emailed to you at an email address that you provide.** Most computers already have PDF viewer software installed, which will allow you to open, read, or print the consent form. The email we send you will include a link to PDF viewer software (such as Adobe Acrobat Reader) in case your computer doesn’t already have it. If you would prefer to receive a paper copy of the consent form at no cost to you, please contact the researcher(s) listed in this consent form.

**The data we collect as part of this research may be used for commercial profit**, such as developing new tests or products. There is no plan to share this profit with you.

A description of this clinical trial will be available on <https://clinicaltrials.gov>, as required by U.S. Law. This Web site will not include information that can identify you. At most, the Web site will include a summary of the results. You can search this Web site at any time.

# What can you do if you want more information?

**Talk to the study team**. We are here to help you understand the study. Please ask us any questions you may have, even about things that are not in this consent form. It is our responsibility to give you the information you need to make a decision and to give you time to think about whether you want to sign up. If you feel you have been harmed by participating, you can contact us about that too.

**Talk to someone else**. If you want to talk about the study with someone who is not part of the study team, talk about your rights as a research subject, or to report problems or complaints about the study, contact the UW Human Subjects Division.

| COVE Study Team | [name] Principal Investigator  [name] Research Scientist | Text/call: [###-###-####]  Call: [###-###-####]  [email address] |
| --- | --- | --- |
| UW Human Subjects Division | | [###-###-####]  [email address] |

**Future Studies consent option:** You may be contacted for other studies in the future. You may choose not to not be contacted in the future.

- No, I do not want to be contacted for future studies.
- Yes, it is okay with me for researchers to contact me for future studies.

**Consent comprehension quiz**:

1. If I am eligible, I will be asked to complete a survey today, complete quick check-ins 3x/day for 2 weeks at the beginning of the study and one week at the end, and then complete a follow-up survey. (True)
2. Depending on which study condition I am assigned to, I might also be invited to complete surveys 3x/day for 1 week during Months 1-11 of this study (True).
3. In addition to surveys, I will definitely receive additional text messages throughout the year. (False)
4. I may be asked to complete an interview about my experience with Caring Contacts but I also might not. (True)
5. My participation is voluntary and I may exit the study without consequence. (True)
6. I will receive Amazon gift codes based on how many procedures I complete. I may be invited to complete more or less surveys, which will affect how much I get paid. If I miss surveys which I receive invites for, I will receive less than the potential amount. If I am invited to participate in an interview, I will receive $50 more. (True)
7. No matter what, I’ll definitely be paid $290 for participating. (False)
8. There are no bonuses for completing more surveys (False)
9. All the information I provide will be confidential, unless the study is reviewed to make sure the research is being done correctly or if there is serious and immediate risk of harm to me or someone else. (True)
10. I am only allowed to enroll for participation one time and will only be enrolled into the study if I meet the eligibility requirements. If the study team believes I have intentionally provided misleading information, it will result in delayed or no payment. (True)

Thanks for answering our questions! Now is the time to let us know whether or not you would like to participate in the study. Please review the following statement:

**By checking yes, I confirm that** the study has been explained to me and I volunteer to participate in the research. I have had a chance to ask questions. If I have questions later about the research or feel I have been harmed by participating in the study, I can contact a member of the research team or the UW Human Subjects Division using the information listed above. I will receive a copy of this consent form.

**Now, please indicate if you consent to participate in the research:**

- Yes, I agree to take part in this study.
- No, I do not want to take part in this study.
