# Supplementary material for: Exploring the Impact of the Caring Contacts Intervention on the Stress and Distress of Veterans and Service Members: Protocol for a Randomized Controlled Trial
Source: JMIR Res Protoc. 2025 Aug 13;14:e72140. doi: 10.2196/72140 (PMC12391844; doi:10.2196/72140)
Supplement: Multimedia Appendix 3 [file resprot_v14i1e72140_app3.docx]

**Client Qualitative Interview guide**

Thank you again for agreeing to complete this last portion of the study with me today. As a reminder, this is a follow up to the Caring Contacts research study that you enrolled in about 3 months ago. We want to learn what it was like to take part in the study. Your answers to these questions will help us to improve future studies and health care for people experiencing stress and distress. There are no right or wrong answers to the questions, I just want to hear about your experience and your perspectives. Negative feedback is at least as helpful as positive!

This should take approximately 30 minutes although we can go longer if you have more you want to share. Please know you can take a break, decline to answer any questions and you can stop the interview at any time. You can also ask to be connected with a counselor at any time.

Before we begin, do you have any questions?

*INTERVIEWER: Ask all questions in bold print below. When the participant does not understand or respond substantively to a question, use the PROBES to elicit additional information. If a participant does not respond to probes with more information, move on to the next question.*

1. **Overall, what has been your experience of participating in this study?**

*Probes:*

1. *What do you remember about the study? Signing up, enrolling, answering surveys, getting messages etc.*
2. *What do you wish had been different, if at all?* *Thinking back, would you change anything about your experience with this study?*
3. **After you enrolled in the study online, you should have received text messages from a member of the study team.**

*Probes:*

1. Opener/context setting - Compared to the other text messages you get day to day, how was it getting these messages?
   1. *What was it like to get these messages?*
   2. *How did the messages make you feel?*
2. How **acceptable** did you find receiving and responding to the messages?
   1. *Message process*
      1. *To what extent did you like or dislike the experience of getting the messages?*
      2. *Who sent the messages?*
   2. *Message content: What kinds of things did the messages say?* *What were the messages about for you?*
      1. *What are some words you would use to describe the content of the messages?*
      2. *What messages were especially meaningful to you, if any?*
3. How **feasible** (e.g., doable) did you find receiving and responding to the messages?
   1. *How well or poor did the timing work for you?*
   2. *Were there aspects of the messages that made it easier or harder for you to respond to?*
4. How **burdensome** was it to receive and respond to messages?
   1. *How was it to get and respond to these messages?*
   2. *How much effort did it take to get and respond?*
5. ***Usability***
   1. How user friendly was it to receive and reply to these messages?
6. *Is there anything else that you especially remember about the messages?*
7. **Did you ever reply to a text message from the study team?**
   1. *[if yes] Continue to Q4.*
   2. *[if no or if they don’t remember] Why not? Skip to Q5.*
8. **Tell me about what that was like.**

*Probes:*

1. *What did you say?*
2. *Did anyone reply to you?*
3. *Was the reply helpful? Why/why not?*
4. *[if no] What would have made the response more helpful?*
5. *Did you ever send a picture or a gif?*
6. **A few months ago, they stopped sending messages due to the study ending.**

*Probes:*

1. *Did you notice any change in the frequency of messages or that they had stopped?*
2. *How did it feel to not get messages?*
3. *Would you change anything about the messages ending (e.g., alerts about the upcoming ending, check in with a researcher)?*
4. *Would you have wanted those messages to keep coming?*
5. *Did your feelings about the study team change once you stopped getting messages? How?*
6. **Over the 3 months you participated in the study, you also received invitations to complete quick check-ins/brief surveys online. How did answering those questions go for you?**

*Probes:*

1. *How did you complete those surveys? (i.e., via phone or computer)*
2. *Did you have any trouble completing the surveys?*
3. *How was the sign-up process for you?*
4. How did it feel to get the survey invites a few weeks after you signed up?
   1. When did you expect to get them?
   2. Did you remember they were going to come/Were you surprised? Why/why not?
5. *About how many surveys do you remember being invited to complete?*
6. *About how many questions do you remember being in each survey?*
7. *About how long do you think it took to complete each survey?*
8. Did you ever miss a survey? How did you feel about that? **How would increasing the time help?**
9. *How do you remember getting the invitations? Did you like the invitations better from one source vs another? (i.e., via text vs. Email)*
10. *How would you compare the longer surveys you did at the beginning and end of the study compared to the brief surveys?*
11. *Anything else you’d like to add about your experience completing those brief surveys?*
12. **Can you tell me how you are feeling about your life now?**

*Probe:*

1. *In what ways, if any, did the messages impact how you feel about your life? What is different compared to before you got the messages?*
2. *What about your sense of the future – in what ways, if any, have you changed your thinking about the future since getting the messages?*

*INTERVIEWER: The remaining questions are lower priority. Proceed with these questions ONLY if the participant is willing to continue or has time.*

**Okay, so since we have a bit more time, I want to focus a little more on the messages themselves.**

1. **How was the timing of the messages? Did any of the messages happen to arrive at a time when it was especially helpful?**
   1. *[if yes] Would you feel comfortable saying what was going on at that time in your life?*
   2. *What about at a time when it was especially unhelpful?*
2. **How did your feelings change about the messages over time?**

*Probe:*

1. *How did you feel about messages when you started receiving them?*
2. *Can you tell me how things changed as time went on?*
3. *What were the reasons for this change?*
4. **If you could change anything about the messages, what would you change?**

*Probe:*

1. *How so? (Content? Timing? Frequency? Size? Cost to you [if charged for texts]? Privacy?)*
2. *Did any messages seem pushy? How so?*
3. **We are always looking for different ways to improve the experience, could I ask your opinion on a few different options?**
   1. *How do you feel about the idea of receiving these messages without being able to respond to them?*
   2. *How do you think you’d feel about the messages being sent* not *by someone you know but automatically, like with a chatbot?*
4. **How often did you interact with the person sending messages other than the messages themselves?**

*Probe:*

1. *Outside of the messages, did you ever reach out to the study team about how you were feeling?*
2. *Did you ever think about contacting someone from the study? Why or why not?*
3. **What suggestions do you have for improving this study for people with stress and distress?**

*Probe:*

1. *Can you tell me more about that?*
2. *In what ways could this program be offered to more people in your community?*
3. **Okay. This is our last question. You have provided some extremely important information which will help us better understand and support people who are going through hard times in their lives. Is there anything else you think we should think about or consider?**
   1. *Probe: What else do we need to know?*
